# The burden and outcomes of stroke in young adults at a tertiary hospital in Tanzania: a comparison with older adults

**DOI:** 10.1186/s12883-020-01793-2

**Published:** 2020-05-25

**Authors:** Sarah Shali Matuja, Patricia Munseri, Khuzeima Khanbhai

**Affiliations:** 1 Department of Internal Medicine, Catholic University of Health and Allied Sciences, Mwanza, Tanzania; 2 Department of Internal Medicine, Muhimbili University of Health and Allied Sciences, Dar es Salaam, Tanzania; 3 Department of Cardiology, Jakaya Kikwete Cardiac Institute, Dar es Salaam, Tanzania

**Keywords:** Stroke, Young adults, Burden, Risks, Hypertension, Fatality, Predictors, Outcomes

## Abstract

**Background:**

Stroke burden in young adults is growing associated with unique risk factors and devastating outcomes. We aimed to investigate the magnitude, risk factors and outcomes of first ever stroke in young adults ≤45 years compared to older adults > 45 years.

**Methods:**

All patients with a World Health Organization clinical definition of stroke at a tertiary hospital in Tanzania were enrolled. The National Institute of Health Stroke Scale and Modified Rankin Scale were used to assess admission stroke severity and outcomes respectively. Kaplan-Meier analysis was used to describe survival and Cox-proportional hazards model was used to examine predictors of fatality.

**Results:**

We enrolled 369 first ever stroke participants over 8 months. First strokes accounted for one quarter of the medical admissions in both younger and older groups, 123/484 {(25.4%) [95% CI 21.5–29.3%]} and 246/919 {(26.8%) [95% CI 23.9–29.6%]} respectively. Hemorrhagic stroke occurred in 47 (42.3%) vs 62 (27.2%) for the young and old respectively *p* = 0.005. Factors associated with stroke in the young were: a new diagnosis of hypertension in 33 (26.8%) vs 23 (9.3%) *p* < 0.001, HIV infection 12 (9.8%) vs 7 (2.8%) *p* = 0.005, use of hormonal contraception in females 33 (48.5%) vs 13 (9.4%) *p* < 0.001, elevated serum low density lipoproteins 28 (27.7%) vs 29 (16.4%) *p* = 0.024, hypercholesteremia 34 (31.2%) vs 40 (20.2%), *p* = 0.031, sickle cell disease 11 (9.7%) vs 9 (4.2%) *p* = 0.047 and thrombocytosis 12 (16.9%) vs 8 (5.6%) *p* = 0.007. The overall 30-day fatality rate was 215 (61.3%); 57 (49.1%) vs 158 (67.2%) in the young and old respectively. Independent predictors of fatality were: severe stroke {HR 10.35 (95% CI: 1.397–76.613)}, leukocytosis {HR 2.23 (95% CI: 1.448–3.419)} and fever {HR 1.79 (95% CI: 1.150–2.776)}.

**Conclusions:**

There is a high burden of stroke in young adults that is coupled with a high 30-day fatality rate. Screening and management of hypertension is crucial in the prevention of stroke. More research is needed to identify factors which cause death, allowing the development of sustainable interventions to reduce early post stroke fatality in this group.

## Background

Stroke is a leading contributor to Disability Adjusted Life Years (DALYs) in low and middle income countries (LMICs), causing 4.85 million deaths and 91.4 million DALYs compared to 1.6 million and 21.5 million deaths and DALYs respectively in high income countries [1]. Stroke incidence increases steeply with age, with a quarter of strokes occurring in individuals below 65 years compared with half occurring in those above 75 years [2]. The population at risk for stroke has increased in LMICs due to improved life expectancy mainly due to the treatment and control of HIV/AIDS [3]. In Tanzania the population life expectancy has increased from 49 years in 1995 to 66 years in 2015 [3].

The increase in the aging population coupled with lifestyle changes has resulted into an increased burden of Diabetes mellitus (DM) and hypertension, two of the main risk factors for stroke [4]. In a three-year Tanzanian population based study conducted between 2004 and 2006, the crude stroke incidence was 107.9 and 94.5 per 100,000 for urban and rural areas respectively and 315.9 and 108.6 per 100,000 respectively after standardizing for age [5]. The authors concluded that there was a higher stroke incidence in urban Tanzania compared to stroke incidence in the developed world. In this study the incidence of stroke was twice as high in individuals below 44 years of age living in urban areas, compared with age matched individuals in rural settings (20.1 Vs 9.0 per 100,000 respectively) [5].

Though stroke is common among the elderly, recent reports indicate a 36% global rise in stroke incidence, (35.1 to 47.6 per 10,000) from 2003 to 2012 among individuals aged between 35 to 44 years [6]. In the United States of America, there has also been a steep increase in stroke incidence in young adults from 22 to 45 per 100,000, mainly seen in African-Americans [7]. In Sub-Saharan Africa (SSA) most of the available data on incidence of stroke in young adults was obtained more than a decade ago with a high incidence of 47 per 100,000 among those under 45 years [8]. However, a more recent study in Nigeria reported a prevalence of 5.4% among those aged between 35 to 44 years compared to 25.6% in the age group between 55 to 64 years [9]. Stroke at a young age paralyzes the nation’s work force that is responsible for maintaining the nation’s economy and population. Interestingly, there has been an increase in risk of ubiquitous vascular factors that were previously thought to be rare in the young population attributed to rapid transitioning [10]. Young adults also have other unique risk factors for stroke in addition to the traditional risk factors of DM and hypertension [10]. The stroke incidence in the young is therefore expected to rise due to the rise in prevalence of hypertension and DM.

We aimed at investigating the magnitude of first ever stroke, the associated risk factors and outcomes at 30 days in young adults compared to older adults at a tertiary hospital in Dar es Salaam, Tanzania.

## Methods

### Study design and population

This cohort study was conducted at a tertiary public teaching hospital, Muhimbili University of Health and Allied Sciences Academic Medical Center (MAMC), in Dar es Salaam, Tanzania. MAMC offers super-specialized medical care for all specialties and receives referrals from public and private hospitals from all over the country as well as walk in patients. We consecutively enrolled consenting participants who were admitted at MAMC with a clinical diagnosis of first ever stroke based on the World Health Organization (WHO) definition for stroke [11]. Participants or their next of kin were required to provide written informed consent and had to be ≥18 years at the time of consent prior to enrollment. Study participants were prospectively enrolled between June 2018 to January 2019 and each participant was followed up for outcomes to 30 days from admission.

### Data collection

An interviewer based structured questionnaire (See Additional file 1: Study questionnaire) was administered to all study participants or their caregivers if the participant was unable to communicate. The questionnaire captured sociodemographic information, mobile numbers, previous stroke risk factors such as history of hypertension, DM, smoking, alcohol consumption, cardiac disease, and HIV infection. Other information collected included: use of medications for hypertension, diabetes, HIV, illicit drugs and use of hormonal contraception for females. The date of onset of stroke symptoms and date of arrival at the hospital were also recorded. Cigarette smoking and alcohol consumption was categorized as ever smoked and taken alcohol in life or never smoked or taken alcohol respectively. Current smokers/current alcohol consumers were defined as cigarette smoking/alcohol consumption within the last 12 months respectively.

All participants had their waist and hip circumference measured using a tape measure and recorded in centimeters. Pulse was checked for rate and rhythm and blood pressure (BP) was measured using a standard digital BP machine, AD Medical Inc. Three BP readings were collected spaced 5 min apart, while the participant was at rest and an average BP was computed. Hypertension was defined as a systolic blood pressure (SBP) ≥140 mmHg or diastolic blood pressure (DBP) ≥90 mmHg. Examination also included measuring temperature, precordial and neck carotid auscultation. All examination findings were recorded in pre-specified case report forms.

We aseptically collected 15mls of venous blood from each study participant: 5mls were analyzed for random total cholesterol, triglycerides, low density and high density lipoproteins using BIO- SYSTEMS machine, 5 mls were analyzed for complete blood count using HEMOLYZER 3 PRO machine and 5mls were analyzed for sickling test. Sickling test was performed using sodium metabisulphite and slides were viewed using Olympus microscope.

Capillary fingertip blood samples were collected from each participant to check for random blood glucose (RBG) levels and HIV rapid testing using a glucometer GLUCOPLUS™ and SD Bioline respectively. Consent for HIV testing was obtained during the initial written informed consent process prior to enrolment. A fasting blood glucose (FBG) sample was collected the following morning for participants with (RBG) levels of ≥11.1 mmol/l. DM diagnosis was defined as a RBG reading of ≥11.1 mmol/l or a FBG reading of ≥7 mmol/l. For participants who were HIV reactive to SD Bioline, were tested using Unigold Biotech.

A non-contrast brain computer tomography (NCCT) using GE Healthcare Optima or magnetic resonance imaging (MRI) using GE SIGNA CREATOR were performed on study participants at the MAMC radiology department and images interpreted by a trained radiologist.

Transthoracic echocardiography (ECHO) using GE Medical Systems was performed by a trained cardiologist and interpretation was based on European Society of Cardiology/American Society of Echocardiography guidelines [12]. Left ventricular muscle mass was assessed using a four chamber view at the end of diastole. A septal thickness > 10 mm and > 11 mm was considered left ventricular hypertrophy (LVH) for females and males respectively. Left atrium (LA) size measurements were performed in m-mode at the end of systole, a diameter > 3.8 cm and > 4.0 cm for females and males respectively was regarded as LA enlargement. Mitral stenosis (MS) was defined as mitral valve area of ≤1.5cm^2^ in short axis view and mean pressure gradient of ≥5 mmHg using continuous wave doppler. A 12-lead electrocardiogram (ECG) using Bionet machine was performed by the principle investigator on the study participants to look for evidence of atrial fibrillation.

Stroke severity was assessed using the National Institute of Health Stroke Scale (NIHSS) [11]. A score of 1–4 was defined as minor stroke, 5–15 moderate stroke, 15–20 moderately severe and 21–42 severe stroke. Stroke outcomes were categorized using the Modified Rankin Scale (MRS) [11] at 24 h, 72 h, 7 days, 14 days and at 30 days from admission, with scores ranging from 0 (no symptoms) to 6 (death). Death of date was recorded at each point and time to event was computed using date difference between the date of last contact (date of death or date of last follow up) and date of admission.

### Data analysis

Data were transferred from the questionnaires in full and entered into SPSS version 20.0 for analysis. The proportions of stroke by age were calculated with 95% confidence intervals. Continuous variables were summarized and presented as means and standard deviation (SD) or medians with Interquartile Range (IQR). Stroke risk factors by age were summarized as proportions and comparisons were made using Pearson’s Chi square test or Fisher’s exact test. Kaplan Meier analysis was used to describe survival probabilities by age groups from date of admission to 30 days. The associations between various patient characteristics with fatality were examined using the Cox-proportional hazards model. Hazard ratios (HR), 95% confidence intervals (CI) and corresponding *p* values were obtained from the models adjusting for potential confounders. Variables with p value < 0.2 in the univariate analysis were included in the multivariate analysis model and significance level was set as a p value of < 0.05.

## Results

### Proportion of stroke admissions

**Fig. 1:**
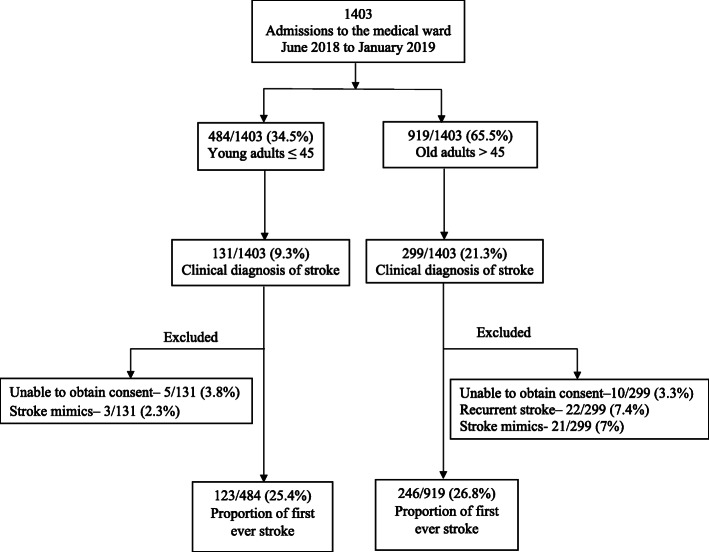
Consort diagram showing the flow of participants

There were a total of 1403 admissions in the medical ward between June 2018 to January 2019, of whom 729 (52%) were females. There were 430 (30.6%) participants who met the WHO definition for clinical stroke; 61 (14.2%) participants were excluded; recurrent stroke 22 (5.1%), stroke mimics 24 (5.6%) and inability to obtain consent 15 (3.5%) as summarized in Fig. 1. This left 369 participants with first ever stroke, of whom 123 were ≤ 45 years (young) and 246 were > 45 years (old). The proportion of stroke compared to total admissions in the young was 25.4% (123/484) {95% CI 21.5–29.3%} and among the old was 26.8% (246/919) {95% CI 23.9–29.6%}.

The overall mean age and SD was 57.4 ± 16 years and was 39.4 ± 5.2 years and 66.4 ± 11.3 years for the young and older stroke participants respectively. The baseline characteristics are summarized in Table 1. The median time from stroke onset to hospital arrival was 2 days IQR [1, 4] in both groups. A total of 146 (39.6%); 55 (44.7%) young vs 91 (36.9%) older, *p* = 0.153 arrived at the hospital within 24 h from stroke symptoms, 183 (49.6%) participants arrived at hospital between day 2 to 6 from onset of symptoms; 57 (46.3%) vs 126 (51.2%) *p* = 0.326 in young and older groups respectively while 40 (10.8%) participants arrived 7 days after stroke symptoms.

**Table 1:** Baseline characteristics of the study participants with stroke

	Age groups		Total *N* = 369 (%)	*P* value
	≤ 45 years *n* = 123 (%)	> 45 years *n* = 246 (%)		
Female	68 (55.3)	138 (56.1)	206 (55.8)	0.882
Marital status				
Ever Married	89 (72.4)	244 (99.2)	333 (90.2)	< 0.001
Never Married	34 (27.6)	2 (0.8)	36 (9.8)	
Residence				
Dar-es-Salaam	92 (74.8)	195 (79.3)	287 (77.8)	0.33
Health Insurance coverage				
Yes	37 (30.1)	65 (26.4)	102 (27.6)	0.459
Alcohol consumption				
Ever	28 (22.8)	45 (18.3)	73 (19.8)	0.309
Never	95 (77.2)	201 (81.7)	296 (80.2)	
Current	23 (18.7)	25 (10.2)	48 (13)	0.022
Cigarette Smoking				
Ever	8 (6.5)	17 (6.9)	25 (6.8)	0.884
Never	115 (93.5)	229 (93.1)	344 (93.2)	
Current	3 (2.4)	12 (4.9)	15 (4.1)	0.263

### Stroke subtypes

Brain imaging was performed on 339 (91.9%) participants, 30 (8.1%) participants did not undergo brain imaging as summarized in Table 2. Young adults were more likely to have hemorrhagic stroke compared to the older participants 47 (42.3%) vs 62 (27.2%), *p* = 0.005 for the young and older groups respectively.

**Table 2:** Brain imaging and stroke subtype in comparison by age groups

CT/MRI findings	Age groups		Total N (%)	*P* value
	≤ 45 years n (%)	> 45 years n (%)		
Missing brain Images	12 (9.8)	18 (7.3)	30 (8.1)	0.419
Died prior to imaging	7 (5.7)	13 (5.3)	20 (5.4)	
Unable to pay for brain imaging	3 (2.4)	3 (1.2)	6 (1.6)	0.722
Technical malfunction	2 (1.6)	2 (0.8)	4 (1.1)	
Stroke sub-type				
Ischemic^a^	51 (45.9)	138 (60.5)	189 (55.8)	0.011
Hemorrhagic^a^	47 (42.3)	62 (27.2)	109 (32.2)	0.005
Mixed lesions ^a^	0 (0.0)	6 (2.6)	6 (1.8)	0.085
Normal ^a^	13 (11.7)	22 (9.6)	35 (10.3)	0.558
Vessel involved (ischemic/mixed lesions)				
Major vessel disease	21 (41.2)	80 (55.6)	101 (51.8)	0.077
Small vessel disease	21 (41.2)	48 (33.3)	69 (35.4)	0.314
Mixed vessel disease	9 (17.6)	16 (11.1)	25 (12.8)	0.23
Area of Hemorrhage (hemorrhage/mixed lesions)				
Intracerebral	42 (89.4)	67 (98.5)	109 (94.8)	0.041
Intraventricular	2 (4.3)	0 (0.0)	2 (1.7)	0.165
Subarachnoid	3 (6.4)	1 (1.5)	4 (3.5)	0.303

^a^Brain imaging performed on 339 participants, 111 were ≤ 45 years and 228 were > 45 years

### Stroke risk factors

Table 3 summarizes factors associated with stroke in the young compared to the old. Those more common in young were, a new diagnosis of hypertension in 33 (26.8%) vs 23 (9.3%) *p* < 0.001, HIV infection 12 (9.8%) vs 7 (2.8%) *p* = 0.005, female use of hormonal contraception 33 (48.5%) vs 13 (9.4%) *p* < 0.001 and illicit drug use 5 (4.1%) vs 2 (0.8%) *p* < 0.044. Mitral stenosis was unique to the young 4 (4%) vs 0 (0%) *p* = 0.013. Other factors more prevalent in the young were sickle cell disease 11(9.7%) vs 9 (4.2%) *p* = 0.047, elevated low density lipoproteins 28 (27.7%) vs 29 (16.4%), *p* = 0.024, hypercholesteremia 34 (31.2%) vs 40 (20.2%), *p* = 0.031 and thrombocytosis 12 (16.9%) vs 8 (5.6%), *p* = 0.007.

**Table 3:** Risk factors for stroke by age groups

Risk factors	Age groups		Total N (%)	*P* value
	≤ 45 years	> 45 years		
	n (%)	n (%)		
Hypertension				
Known	78 (63.4)	207 (84.1)	285 (77.2)	< 0.001
New diagnosis	33 (26.8)	23 (9.3)	56 (15.2)	< 0.001
On treatment	30 (38.5)	88 (42.5)	118 (41.4)	0.536
Diabetes				
Known	17 (13.8)	47 (19.1)	64 (17.3)	0.206
New diagnosis	2 (1.6)	1 (0.4)	3 (0.81)	0.259
On treatment	11 (64.7)	20 (42.6)	31 (48.4)	0.117
HIV infection				
Known	12 (9.8)	7 (2.8)	19 (5.1)	0.005
New diagnosis	2 (1.6)	5 (2)	7 (1.9)	1
On treatment	11 (91.7)	7 (100)	18 (94.7)	1
Hormonal contraception	33 (48.5)	13 (9.4)	46 (22.3)	< 0.001
Illicit drugs	5 (4.1)	2 (0.8)	7 (1.9)	0.044
Family history of hypertension	42 (34.1)	83 (33.7)	125 (33.9)	0.938
Family history of diabetes	11 (8.9)	33 (13.4)	44 (11.9)	0.211
Family history of sudden death	8 (6.5)	11 (4.5)	19 (5.1)	0.405
Mid diastolic murmur	4 (3.3)	0 (0)	4 (1.1)	0.012
Increased waist- hip ratio	95 (77.2)	197 (80.1)	292 (79.1)	0.526
Hypercholesteremia	34 (31.2)	40 (20.2)	74 (24.1)	0.031
Elevated low density lipoproteins	28 (27.7)	29 (16.4)	57 (20.5)	0.024
Sickle cell	11 (9.7)	9 (4.2)	20 (6.1)	0.047
Thrombocytosis	12 (16.9)	8 (5.6)	20 (9.3)	0.007
ECG findings				
Atrial fibrillation	3 (3)	18 (9)	21 (7)	0.059
ECHO findings				
Mitral stenosis	4 (4)	0 (0)	4 (1.4)	0.013
Left ventricular hypertrophy	76 (76)	173 (90.6)	249 (85.6)	0.001

### Stroke severity

A total of 207 (56.1%) participants met the definition for severe stroke on admission based on the NIHSS, 54 (43.9%) vs 153 (62.2%), *p* < 0.001 for the young and older participants respectively. Moderate stroke occurred in 44 (35.8%) vs 60 (24.4%), *p* = 0.022 for the young and older participants respectively.

### Stroke fatality

**Fig. 2:**
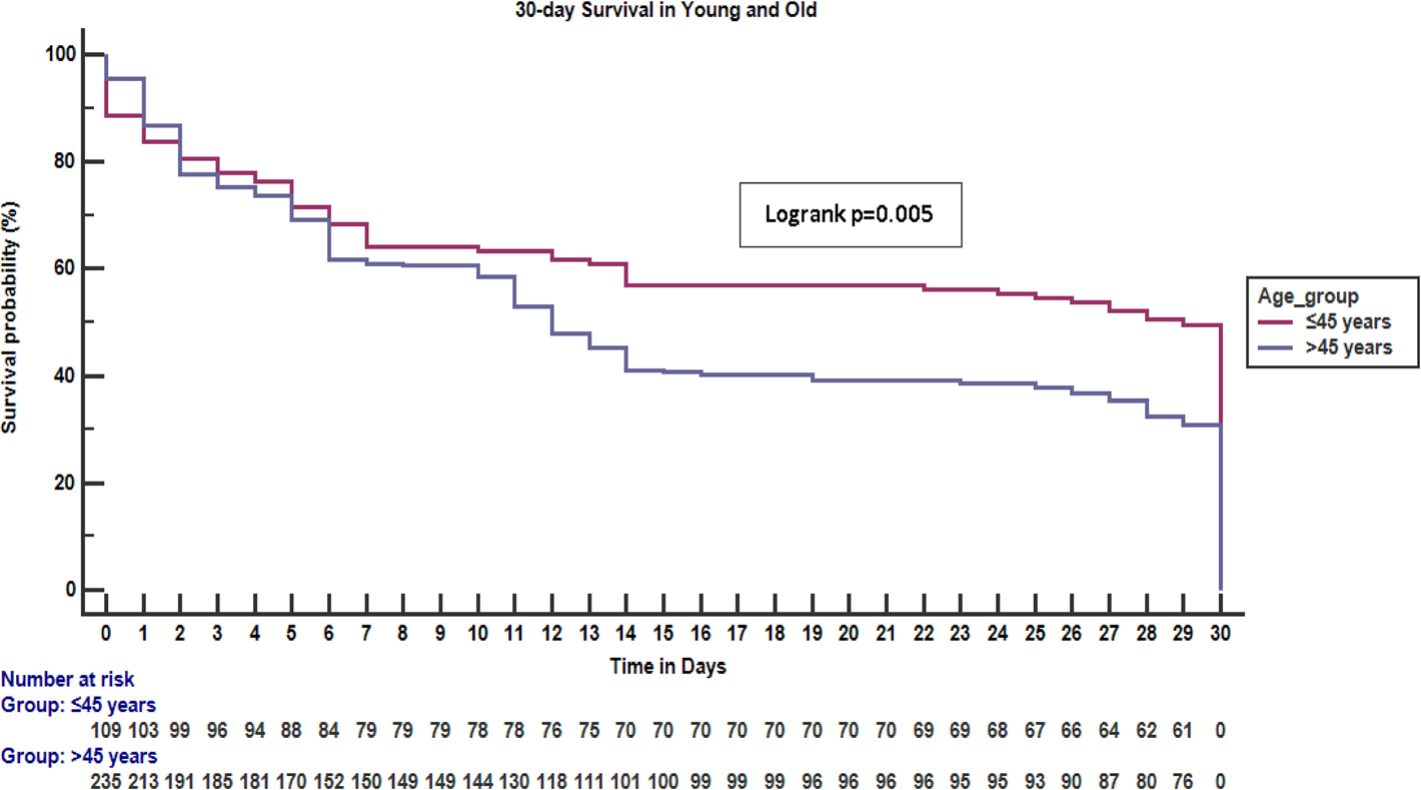
Kaplan Meier survival curve for 30-day survival in young and old stroke participants

The overall fatality rate at 30 days was 215 (61.3%); 57 (49.1%) vs 158 (67.2%), *p* = 0.001 in the young and old respectively. Fatality was highest within the first week of hospital admission as summarized in Fig. 2, Kaplan Meier curve for 30-day survival in young and old stroke participants. There were 18 (4.9%) participants who were lost to follow up following discharge from the hospital 7 (5.7%) young and 11 (4.5%) old, *p* = 0.608.

### Predictors of fatality

The predictors of fatality are summarized in Table 4. In univariate analysis, factors that were associated with increased 30-day case fatality were: male gender, older age > 45 years, DM, HIV infection, severe stroke, tachycardia, bradycardia, atrial fibrillation, fever and leukocytosis. In multivariate analysis after adjusting for other factors, independent predictors of 30-day fatality were: severe stroke {HR 10.35 (95% CI: 1.397–76.613)}, leukocytosis {HR 2.23 (95% CI: 1.448–3.419)} and fever {HR 1.79 (95% CI: 1.150–2.776)}.

**Table 4:** Predictors of fatality

Predictor	Unadjusted HR (95% CI)	*P* value	Adjusted HR (95% CI)	*P* value
Gender				
Female	1		1	
Male	1.27 (0.97–1.66)	0.079	1.29 (0.85–1.97)	0.232
Age groups				
≤ 45 years	1		1	
> 45 years	1.53 (1.13–2.07)	0.006	1.11 (0.690–1.77)	0.676
Hypertension				
No	1			
Yes	1.07 (0.63–1.81)	0.805		
Diabetes mellitus				
No	1		1	
Yes	1.36 (0.87–1.90)	0.077	1.03 (0.63–1.70)	0.896
HIV infection				
No	1		1	
Yes	1.48 (0.92–2.40)	0.111	2.32 (0.91–5.90)	0.079
Stroke severity				
Minor	1		1	
Moderate	1.33 (0.41–4.31)	0.632	2.51 (0.34–18.78)	0.37
Severe	7.45 (2.37–23.38)	0.001	10.35 (1.39–76.61)	0.02
Pulse rate				
Normal	1		1	
Tachycardia	1.62 (1.21–2.18)	0.001	0.861 (0.57–1.29)	0.474
Bradycardia	1.74 (0.83–3.63)	0.14	1.051 (0.37–3.02)	0.927
Atrial fibrillation				
No	1		1	
Yes	1.77 (1.05–2.96)	0.032	1.34 (0.71–2.54)	0.366
Fever				
No	1		1	
Yes	2.61 (1.98–3.45)	< 0.001	1.79 (1.15–2.78)	0.01
Leukocytosis				
No	1		1	
Yes	3.03 (2.07–4.44)	< 0.001	2.23 (1.45–3.42)	< 0.001
Anemia				
No	1			
Yes	1.17 (0.82–1.66)	0.383		
Thrombocytopenia				
No	1			
Yes	1.27 (0.86–1.89)	0.23		
Stroke subtype				
Mixed	1			
Ischemic	0.97 (0.36–2.63)	0.956		
Hemorrhagic	1.16 (0.42–3.18)	0.774		

*HR* Hazard ratio

## Discussion

There is a high proportion of first ever stroke cases in young adults, with similar rates observed to that of the older population admitted at MAMC. These findings are quite alarming as stroke was initially thought to be a disease of the elderly with incidence rates expected to increase incrementally with age. The Global Burden of Disease report stated that stroke should no longer be regarded as a disease of the old as it has the same potential of affecting the young [13]. This high stroke burden in the young calls for urgent interventions that should address risk factors as approximately 50% of the Tanzanian population comprises of individuals between 15 to 54 years who are the nation’s work force [14].

The high stroke rates in young adults observed in our study was attributed to several factors such as hypertension. Hypertension accounted for more than 90% of all stroke admissions and a new diagnosis of hypertension was statistically more prevalent in the young compared to the older counterparts. However, the high rates of new hypertension could be a reflection of the initial sympathomimetic effect in acute stroke to maintain cerebral autoregulation [15], however it is notable that three-quarters of the young participants had evidence of left ventricular hypertrophy on echocardiography, suggesting a more chronic problem. Hypertension is a known risk factor for stroke in young adults similar to what has been reported previously [16]. Our results also suggest that hypertension in young adults is untreated as only 38.5% of the young strokes with premorbid hypertension were on regular treatment. This indeed calls for awareness and regular screening for hypertension as observed in four SSA countries whereby 45% of all strokes could be prevented by simply measuring and controlling blood pressure [17]. Further studies are recommended on etiologies of hypertension among young adults in Tanzania.

We observed a significant contribution of hemorrhagic stroke in the young. In India hemorrhagic stroke secondary to hypertension was common in the young [16]. Hypertension causes lipohyalinosis and micro-atheroma that results in rupture of cerebral blood vessels [18].

Mitral stenosis was a unique risk factor for stroke in the young mainly due to atrial fibrillation with consequent intracardiac thrombus formation that eventually dislodges to the brain. Prevention of rheumatic heart disease in the young is an important strategy. Use of anticoagulant therapy to prevent thrombus formation and valvular repair is warranted for patients with mitral stenosis.

HIV infection was common in the young. HIV itself or with associated opportunistic infections results in vasculitis, cardio-embolism and coagulopathy [19]. Likewise, the use of certain antiretroviral (ARV) therapy for HIV has been known to cause dyslipidemia that predisposes to stroke [20]. ARVs also prolong patient survival thus predisposing individuals to traditional risk factors for stroke.

The use of oral contraceptives among young females with stroke was another factor observed in this study. Combined oral contraceptives increase the likelihood of ischemic stroke by causing thrombosis especially in women who smoke and have hypercholesteremia [21]. There is a need to screen women for preexisting risks prior to prescribing oral contraceptive pills.

Sickle cell disease (SCD) was another risk factor observed among the young in our study as described previously especially in individuals below 35 years [22]. In this study we did not look into specific sickle cell genotypes, however we propose regular follow up of SCD patients with the use of drugs such as hydroxyurea so as to prevent cerebrovascular complications.

A significant proportion of young adults had hypercholesteremia and increased LDL. Dyslipidemia is a known risk factor for stroke by promoting atherosclerosis which in turn impairs blood pressure regulation predisposing individuals to hypertension [16]. Similarly, factors such as smoking, alcohol consumption and diabetes mellitus which were observed in the current study are also known to exacerbate the likelihood of dyslipidemia. We thus recommend screening for dyslipidemia in young adults and promoting lifestyle modifications.

There was an alarming overall high fatality rate of 61% at 30 days, with high rates in both the young and old. The WHO report for 2017, indicated that stroke is the 6th top cause of death in Tanzania, however mortality data for stroke in the young were lacking [23]. This study has shed some light on the contribution of stroke deaths among young adults. Furthermore, the current study has a high fatality compared to what was reported 2 years ago at a tertiary hospital in Tanzania that reported a 30-day fatality rate of 33.3% [24]. One of the strongest predictors of 30-day fatality observed in this study was severe stroke on admission, based on the NIHSS. Severe stroke is associated with poor outcomes as previously described [25, 26]. Further, it has been reported that any additional point to the baseline NIHSS score decreases the likelihood of favorable outcomes at 7 days by 24% [26]. Other predictors of fatality included presence of fever and leukocytosis, which serve as clinical and laboratory makers for probable infections. Fever after stroke can also be endogenous, usually referred to as central fever, but regardless of the cause it is associated with poor outcomes [27]. Though our study did not look into causes of death, infections such aspiration pneumonia and sepsis have been reported as leading causes of death following stroke [24, 28]. This calls for development of multi-disciplinary stroke units to ensure optimal care to all stroke patients and prevention of post stroke complications.

There are limitations to our study; we only studied the short term post-stroke outcomes and there was limited data available on the causes of death. We recommend a long term follow up of stroke in the young and studies investigating the causes of early fatality. This was a single center study therefore the results may not reflect the general population.

## Conclusions

There is a high burden of stroke in young adults that is coupled with a high 30-day fatality rate. Screening and management of hypertension is crucial in the prevention of stroke. Severe stroke, leukocytosis and fever were independent predictors of case fatality. More research is needed to identify factors which cause death, allowing the development of sustainable interventions to reduce early post stroke fatality in this group.

## Supplementary information

**Additional file 1.** Study questionnaire.

## Data Availability

Dataset used for analysis in this study is not publicly available to maintain participant confidentiality. Data are however available from the corresponding author on reasonable request.

## Abbreviations

AF: Atrial Fibrillation
ADC: Apparent Diffusion Coefficient
DALYS: Disability Adjusted Life Years
DWI: Diffusion Weighted Image
FBG: Fasting Blood Glucose
HIV: Human Deficiency Virus
MRS: Modified Rankin Scale
NIHSS: National Institute of Health Stroke Scale
RHD: Rheumatic Heart Disease
SSA: Sub Saharan Africa
WHO: World Health Organization
