# Supplementary material for: The burden and outcomes of stroke in young adults at a tertiary hospital in Tanzania: a comparison with older adults
Source: BMC Neurol. 2020 May 25;20:206. doi: 10.1186/s12883-020-01793-2 (PMC7247244; doi:10.1186/s12883-020-01793-2)
Supplement: Supplementary file 1 — Additional file 1. Study questionnaire. [file 12883_2020_1793_MOESM1_ESM.docx]

**Additional file 1: Study questionnaire**

**Demographic information case report form**

- Questionnaire no. ..........................
- Hospital registration no. .............................
- Patient’s initials .......................................
- Date of study enrollment .........................
- Date of admission ......................................
- Date of birth ..................................................
- Gender Male Female
- Physical address .............................................
- Marital status

Ever married Never married/single

- Possession of health insurance

Yes No

- Mobile numbers

Patient’s mobile number ......................................................

Next of Kin’s mobile number ..................................................

Close Relative mobile number .........................................................

- Date of stroke symptoms .................................................

**Previous stroke risk factors case report form**

**(**Tick were appropriate) answered by the patient or relative

1. Have you ever been diagnosed for hypertension?

Yes No I don’t know

*If No or I don’t know, skip to question 3.

1. If yes, are you on any regular anti-hypertensive medications?

Yes No

1. Any family history of hypertension?

Yes No I don’t know

1. Have you ever been diagnosed for diabetes?

Yes No I don’t know

*If No or I don’t know, skip to question 6.

1. If yes, are you on any regular oral hypoglycemic drugs or insulin injections?

Yes No

1. Any family history of diabetes?

Yes No I don’t know

1. Any history of hormonal contraception use? (for a female patient)

Yes No

1. Any history of illicit drug use?

Yes No

1. Any history of cardiac diseases?

Yes No I don’t know

If Yes, specify .................................................................

1. Any history of cardiac related surgeries (valvular replacement)?

Yes No

1. Are you HIV infected?

Yes No I don’t know (Never tested)

*If No or I don’t know, skip to question 13.

1. If yes, are you on any regular ARVs?

Yes No I don’t know

1. Are you a cigarette smoker?

Yes No I don’t know

*If No skip to question 15.

1. If yes, are you a current smoker? (smoked within last 12 months)

Yes No I don’t know

1. Do you drink alcohol?

Yes No I don’t know

1. If yes, are you a current consumer? (consumed within 12 months)

Yes No I don’t know

**Clinical assessment case report form**

- Stroke severity on admission case report form using the National Institute of Health Stroke Scale (11).
- Blood pressure

| Time | 1^st^ Reading | 2^nd^ reading | 3^rd^ reading | Average |
| --- | --- | --- | --- | --- |
| On admission |  |  |  |  |

- Pulse rate .................. b/min
  - Rhythm ....................................
  - Heart rate .................................... b/min
  - Pulse deficit ........................... b/min
- Temperature ............. ⁰C
- Carotid bruit - 1. Yes 2. No

- Mid diastolic murmur auscultated- 1. Yes 2. No

- Waist circumference ................................. cm
- Hip circumference ...................................... cm
- Waist-hip ratio ..............................................

**Laboratory investigations case report form**

- RBG ................. mmol/l FBG ................. mmol/l

- HIV test 1. Reactive 2. Non-reactive 3. Indeterminate

- Sickling Test 1. Positive 2. Negative

- Lipid profile

Total cholesterol .............mg/dl

LDL ................................. mg/dl

HDL ............................... mg/dl

TGA ............................... mg/dl

- Complete blood count

Total WBC ......................................... *10^9^/L

Hemoglobin ........................... g/dl

Platelet count ............................... *10^9^/L

**ECG case report form**

Heart rate:

Rhythm:

Presence of p waves:

Present

Absent

**Echocardiography case report form**

- Mean diastolic septal thickness ........................... mm
- Mitral stenosis Present Absent

- If present, Mitral valve area ..................................... cm^2^
- Mean Mitral Valve Pressure Gradient ................................... mmHg
- LA size ............................................ cm
- LA thrombus Present Absent

- If present, size of the thrombus .............................. cm
- Vegetations on the mitral valve Present Absent

- If present, size of the vegetation .......................... cm

**Stroke subtype case report form**

Date of CT scan .........................................

- Normal
- Ischemic
- Large vessel disease
- Small vessel disease
- Hemorrhagic
- ICH
- IVH
- SAH
- Other findings, specify ...............................................

**Stroke outcomes case report form**

Using the Modified Rankin Scale (11) used at 24 hours, 72 hours, day 7, day 14 and 30 days’ post stroke.

Date of death ........................................

Date of discharge .....................................
